# Small Molecule–Peptide Conjugates as Dimerization Inhibitors of *Leishmania infantum* Trypanothione Disulfide Reductase

**DOI:** 10.3390/ph14070689

**Published:** 2021-07-17

**Authors:** Alejandro Revuelto, Isabel López-Martín, Héctor de Lucio, Juan Carlos García-Soriano, Nicola Zanda, Sonia de Castro, Federico Gago, Antonio Jiménez-Ruiz, Sonsoles Velázquez, María-José Camarasa

**Affiliations:** 1 Instituto de Química Médica (IQM-CSIC), E-28006 Madrid, Spain; alejandro_revu@hotmail.com (A.R.); isabelopezmartin@gmail.com (I.L.-M.); nicoh41@gmail.com (N.Z.); sonia@iqm.csic.es (S.d.C.); 2 Departamento de Biología de Sistemas, Universidad de Alcalá, E-28805 Alcalá de Henares, Spain; hector.lucio@edu.uah.es (H.d.L.); jcarlos.garcias@uah.es (J.C.G.-S.); antonio.jimenez@uah.es (A.J.-R.); 3 Unidad Asociada al IQM-CSIC, Área de Farmacología, Departamento de Ciencias Biomédicas, Universidad de Alcalá, E-28805 Alcalá de Henares, Spain; federico.gago@uah.es

**Keywords:** leishmaniasis, small molecule–peptide conjugates, dimerization inhibitors, trypanothione disulfide reductase

## Abstract

Trypanothione disulfide reductase (TryR) is an essential homodimeric enzyme of trypanosomatid parasites that has been validated as a drug target to fight human infections. Using peptides and peptidomimetics, we previously obtained proof of concept that disrupting protein–protein interactions at the dimer interface of *Leishmania infantum* TryR *(Li*TryR*)* offered an innovative and so far unexploited opportunity for the development of novel antileishmanial agents. Now, we show that linking our previous peptide prototype **TRL38** to selected hydrophobic moieties provides a novel series of small-molecule–peptide conjugates that behave as good inhibitors of both *Li*TryR activity and dimerization.

## 1. Introduction

Leishmaniasis is a vector-borne protozoan infectious disease that affects around 12 million people and falls within the category of neglected tropical diseases [1]. It is one of the leading causes of mortality and morbidity in about 100 countries of mainly tropical and subtropical areas, but also in Mediterranean countries [1,2,3,4]. There are more than twenty *Leishmania* species that cause different forms of leishmaniasis. Among them, *L. infantum* and *L. donovani*, which are transmitted through the bite of an infected female sand fly (*Phlebotomus*), are responsible for visceral leishmaniasis, one of the most severe forms of the disease in human beings that causes 26,000–60,000 deaths each year [1]. 

Currently, the lack of a vaccine makes the control of leishmaniasis highly dependent on costly drug therapy [5,6]. There are a limited number of drugs including pentavalent antimonials, pentamidine, amphotericin B, paromomycin, and miltefosine, all of which can have severe side effects, host toxicity, emergence of resistance, and/or administration problems [7,8,9,10]. Therefore, there is a pressing need for the development of more efficient and safer drugs to treat this parasitic disease.

Trypanothione disulfide reductase (TryR), an essential flavoprotein of *Leishmania* parasites [1,11], is a homodimeric enzyme crucial for antioxidant defense against trypanosomatids, and it is absent in humans. TryR catalyzes the NADPH-dependent reduction of trypanothione disulfide (TS_2_) to the corresponding dithiol (T[SH]_2_) [12,13]. The cellular role of TryR and T[SH]_2_ is to maintain intracellular redox equilibrium, in a similar manner to that exerted by the glutathione-glutathione disulfide reductase pair in mammals [14]. TryR is a validated therapeutic target for the discovery of antileishmanial drugs [15,16,17]. The majority of TryR enzyme inhibitors described in the literature interact at the active site where TS_2_ binds. One of the major disadvantages of this approach is that the binding affinity of these competitive inhibitors must be very high because parasite survival is only compromised when the activity of this enzyme is reduced by more than 90% [18], which has turned out to be a very elusive goal in vivo. Therefore, noncompetitive, allosteric, or irreversible inhibitors are very attractive, albeit also challenging, alternatives [19,20,21].

Considering that the biologically functional form of TryR is a homodimer, in 2013, we devised an alternative inhibition strategy aimed at disrupting the protein–protein interactions at the homodimer interface of *Leishmania infantum* TryR (*Li*TryR) [22]. Thus, by combining molecular modeling and site-directed mutagenesis studies, we identified and validated, for the first time, Glu436 (located at an interfacial α-helix spanning from Pro435 to Met447) as a “hotspot” for *Li*TryR homodimer stabilization [22]. As a “proof of concept”, we designed and tested a small library of linear peptides [22] that were rational variations of the P435-M447 α-helix, which includes the dimerization hotspot Glu436. Among them, the *N*^α^-acetylated 13-mer peptide Ac-PKIIQSVGISMKM-NH_2_ (**TRL35**) was found to be a potent enzyme dimerization disruptor and a strong inhibitor (in the submicromolar range) of the oxidoreductase activity of *Li*TryR [22]. The C-terminal truncated peptide Ac-PKIIQSVGI-NH_2_ (**TRL38**) was identified as the shortest peptide sequence that showed inhibition of *Li*TryR oxidoreductase activity and also the ability to disrupt enzyme homodimerization [22]. With a view to optimizing these novel *Li*TryR inhibitors and developing peptidomimetics with enhanced proteolytic stability, a variety of helix-stabilized cyclic and stapled peptides [23,24,25], as well as α,β^3^-peptide foldamers [26], were prepared thereafter. All these peptides and peptidomimetics required attachment to cell-penetrating peptides to allow their cellular uptake and subsequent killing of the parasites in cell culture [25,26]. To overcome the unfavorable drug-like properties of these early prototypes, our efforts were then directed towards the design of small molecules (pyrrolopyrimidine-, imidazole-, and triazole-phenyl-thiazole scaffolds) endowed with high potency and more favorable pharmacokinetic characteristics [27,28]. Nonetheless, our interest in peptide-based compounds did not wane because these types of compounds are gaining momentum as innovative therapies against different diseases, including leishmaniasis [29,30], due to their biocompatibility, versatility, low toxicity, and concise synthesis [30,31]. 

**Figure 1:**
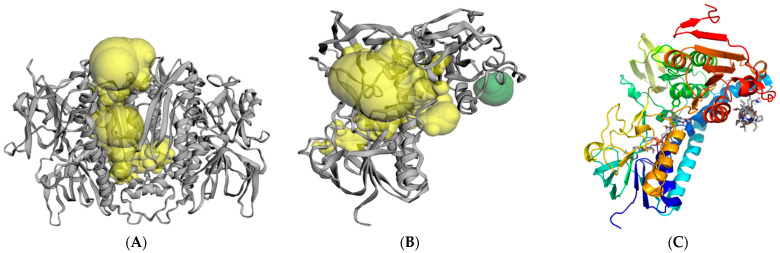
(**A**) An illustration of the *Li*TryR dimer (PDB entry 6I7N [27]) showing the large channel (yellow) connecting the NADPH entry site to the interfacial cavity [28], as calculated by the CASTp web server [32]; (**B**) an illustration of a *Li*TryR monomer showing a similar channel (yellow, 2016 Å^2^, 2550 Å^3^) plus a second one (green, 129 Å^2^, 125 Å^3^) that makes up the putative target site for the dimer-disrupting peptides and SMPCs; (**C**) theoretical model of the complex between prototype peptide **TRL38** (C atoms in grey) and a *Li*TryR monomer (rainbow-colored illustration, with *N*- and C-termini in blue and red, respectively).

Therefore, as part of our continuing efforts to develop novel peptide-based dimerization disruptors of *Li*TryR, we tried to improve the binding affinity of our previously reported inhibitory peptides by linking them to selected hydrophobic moieties. For the design of these small molecule–peptide conjugates (SMPCs), first, we characterized the putative binding of the peptide prototype **TRL38** to a *Li*TryR monomer (Figure 1) using computational methods, and then identified adjacent regions that could additionally be targeted. Our results highlighted the existence of two large hydrophobic pockets lined by Phe396, Pro398, and Leu399 located at ~5 Å from the *N*-terminus of the peptide (Figure 1A).

**Figure 2:**
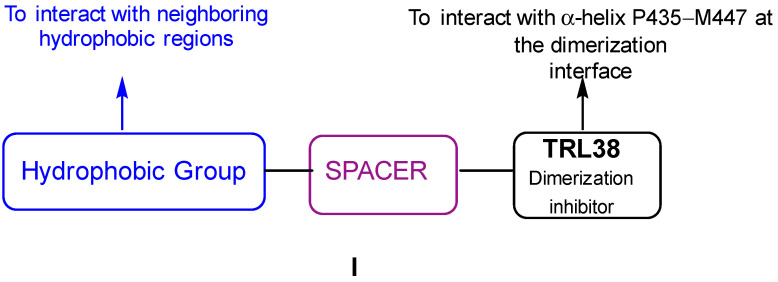
General structure of the novel small molecule **TRL38** conjugate.

Consequently, we decided to synthesize and test a series of SMPCs of general structure **I** (Figure 2) in which the bulky nonpolar moieties attached to the N-terminus of **TRL38** were meant to interact with hydrophobic regions close to the targeted interfacial α-helix by means of selected spacers of different lengths and physico-chemical properties.

## 2. Results

### 2.1. Synthesis

**Scheme 1:**
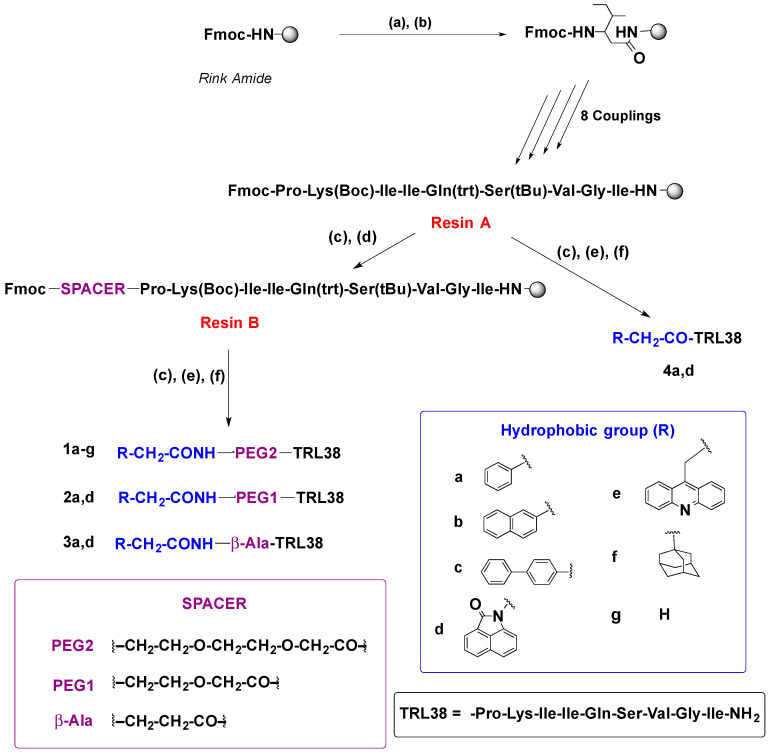
Solid-phase synthesis of hydrophobic SMPCs **1a**–**g**, **2a**,**d**, **3a**,**d**, and **4a**,**d** by amide coupling. Reagents and conditions: (**a**) 20% Piperidine/DMF; (**b**) Fmoc-Ile-OH, HCTU, DIPEA, DMF, MW, 40 °C, 3 x 10 min; (**c**) DBU/piperidine/DMF; (**d**) Fmoc-SPACER-OH, HCTU, DIPEA, DMF, rt, 2h; (**e**) R-CH_2_-COOH, HCTU, DIPEA, DMF, rt, 2 h; (**f**) TFA, TIPS, and H_2_O.

First, a series of dimerization disruptors containing peptide **TRL38** linked to different hydrophobic groups linked to a flexible polyethylene glycol (PEG2) spacer through an amide-type bond (Scheme 1, **1a**–**g**) were prepared based on the premise that the PEG2 linker should improve the water solubility of the final compounds. To study the optimum length of the spacer for activity, a second series of derivatives bearing either a phenyl ring (model compound **2a**) or a tricyclic lactam (compound **2d**) as the hydrophobic substituent bonded to **TRL38** through a shorter polyethylene glycol (PEG1) linker were synthesized (compound **1d** showed the best biological results in the first series of compounds). Since β-alanine was also envisaged as a shorter linker, compounds **3a** and **3d**, bearing the same substituents as **2a** and **2d**, were also prepared. Finally, for comparative purposes, we synthesized compounds **4a** and **4d**, in which the hydrophobic groups were directly linked by means of an amide bond to the *N*-terminus of **TRL38** (Scheme 1).

All the SMPCs bearing an amide-bonded substituent were prepared manually following standard solid-phase peptide synthesis (SPPS) protocols using a Rink amide MBHA polystyrene resin and Fmoc/^t^Bu chemistry. *O*-(1*H*-6-chlorobenzotriazole-1-yl)-1,1,3,3-tetramethyluronium hexafluorophosphate (HCTU) [33] was employed as the coupling reagent for peptide elongation, followed by linking of the appropriate spacers to the corresponding R-CH_2_-COOH acids. The substituted peptides were isolated as carboxamides at the C-terminus. 

The synthesis of **1a**–**g**, **2a**,**d**, **3a**,**d**, and **4a**,**d** was carried out as follows: After Fmoc deprotection of the resin, under standard piperidine conditions, the first amino acid was introduced in the presence of HCTU as the coupling reagent and DIPEA as the base, at room temperature for 2 h. The coupling of the following amino acids (the side chains of Ser protected with ^t^Bu, Gln with Trt, or Lys with Boc groups) was carried out under microwave irradiation at 40 °C in the presence of HCTU/DIPA (3 × 10 min). Upon completing elongation, the Fmoc-group of the *N*-terminus of the peptide (RESIN A) was deprotected, and then the appropriate spacers were coupled (HCTU, DIPEA, DMF room temperature for 2 h), followed by Fmoc deprotection and treatment with the corresponding acids (HCTU, DIPEA, and DMF, room temperature for 2 h) to give, after final Fmoc deprotection and cleavage (TFA, TIPS, and H_2_O), the substituted SMPCs **1a**–**g**, **2a**,**d** and **3a**,**d** (Scheme 1). In addition, piperidine deprotection of the N-terminal Fmoc-group of RESIN A, followed by coupling with commercially available 2-oxobenzo[*cd*]indol-1(2*H*)-yl acetic acid and 3-(acridin-9-yl)propionic acid, using HCTU as the coupling reagent, gave the final peptide conjugates **4a**,**d** (Scheme 1) after deprotection and TFA cleavage from the resin.

**Scheme 2:**
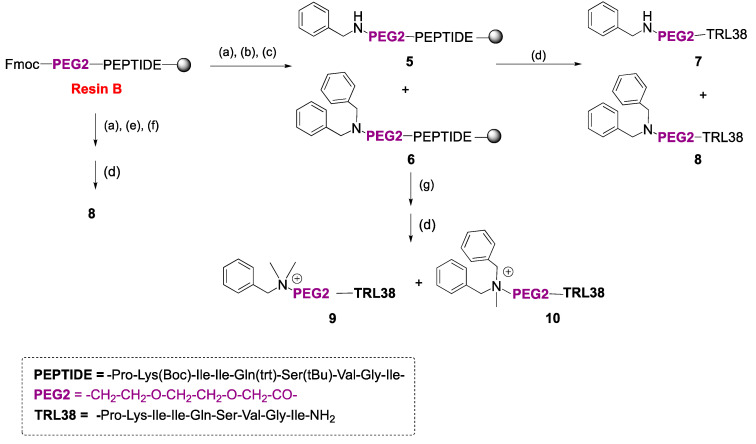
Solid-phase synthesis of hydrophobic SMPCs **7**–**10** by reductive amination followed by amine quaternization. Reagents and conditions: (**a**) DBU, piperidine, DMF; (**b**) PhCHO (3.6 equivalents); (**c**) NaBH_3_CN (3.6 equivalents); (**d**) TFA, TIPS, H_2_O; (**e**) PhCHO (10 equivalents); (**f**) NaBH_3_CN (10 equivalents); (**g**) MeI, 2,6-lutidine.

We were also interested in synthesizing and testing SMPCs **7** and **8** (Scheme 2), in which the hydrophobic group is bonded to the linker through an aminoalkyl-type bond. These derivatives would allow us to study the influence of the nature of the linkage (amide- vs. aminoalkyl-type) between hydrophobic groups and spacers, and also to obtain positively charged SMPCs (**9** and **10**, Scheme 2) that might eventually give rise to additional interactions with negatively charged amino acids, close to the putative binding site at the *Li*TryR dimer interface.

The synthesis of **7** and **8** was performed by solid phase reductive amination [34]. Thus, treatment of Fmoc-PEG2-peptidyl RESIN B (Scheme 2) with DBU and piperidine in DMF, followed by reductive amination reaction with 3.6 equivalents of benzaldehyde and NaCNBH_3_ (3.6 equivalents), afforded, upon cleavage from the resin (TFA, TIPS, H_2_O), a mixture of mono- and di-aminoalkylated **7** and **8**, in 32% and 9% yield, respectively, after purification. However, the disubstituted SMPC **8** was exclusively obtained in 21% yield when ten equivalents of benzaldehyde and NaCNBH_3_ were used instead. Finally, treatment of the mixture of **5** and **6** with MeI and 2,6-lutidine provided the quaternized SMPCs **9** and **10** in 25% and 8% yield, respectively, after cleavage and purification. All the compounds synthesized in this work were characterized as trifluoroacetate salts by analytical HPLC and HRMS after purification on a Biotage Isolera or by semi-preparative HPLC.

### 2.2. Biological Evaluation

All the synthesized novel SMPCs were evaluated as *Li*TryR inhibitors in both the oxidoreductase activity [35] and the dimer quantitation assays [22,35]. The prototype peptide **TRL38** was included as the reference compound. The IC_50_ values obtained for activity (${\mathrm{IC}}_{50}^{\mathrm{act}}$) and dimerization (${\mathrm{IC}}_{50}^{\dim}$) are shown in Table 1.

### 2.3. Molecular Modeling and Computer Simulations

Characterization of the geometric and topological properties of *Li*TryR dimer and monomer by means of the CASTp server [32] showed the largest pocket connecting the bulk solvent to the isoalloxazine ring of the FAD cofactor (Figure 1) and delineated the cavity at one monomer that makes up the binding site for the P435─M447 α-helix from the other monomer.

**Figure 3:**
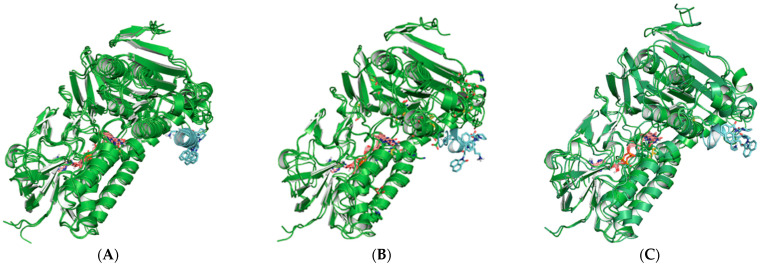
Time evolution of the simulated complex between *Li*TryR monomer (C atoms in green) and SMPC **4d** (C atoms in cyan). The superimposed snapshots correspond to times 5, 25, and 50 ns (**A**), 75 and 100 ns (**B**), and 125 and 150 ns (**C**) of the unrestrained molecular dynamics trajectory in explicit solvent. The FAD cofactor is displayed as sticks with C atoms colored in pink.

Molecular dynamic simulations of the putative complex formed between a *Li*TryR monomer and **4d**, taken as a representative linkerless SMPC, showed initial preservation of the α-helical conformation of the peptide, but subsequent disruption due to protein disorganization at the interfacial region encompassing G448—S470 and mutual side chain adaptation (Figure 3). This progression is likely responsible for dimer disruption by shifting the monomer-dimer conformational equilibrium towards the monomeric, inactive form. Although the naphtholactam moiety initially stabilized the α-helix by means of hydrophobic interactions with the Pro and Ile side chains present at the *N*-terminus of the peptide (Figure 3A), these contacts were later lost and eventually exchanged with interactions with other residues (Figure 3C). A similar behavior can be expected for **1d**, **2d**, and **3d** possessing longer linkers, in view of the experimental finding that the impact of linker length on activity appears to be almost negligible.

## 3. Discussion

In general, all the hydrophobic moiety **TRL38** conjugates were good inhibitors of both activity and dimerization of the enzyme, showing a significant improvement with respect to the parent **TRL38** peptide, regardless of the kind of linkage (i.e., amide- or aminoalkyl-type) between the spacer and the hydrophobic group. This enhancement is highly relevant for SMPCs **1a**, **1d**, **1f**, **2d**, **3d**, **4d**, **7**, and **8**. The length and nature of the spacer does not appear to have a significant influence on activity/dimerization of the compounds (cf., for example, **1d** vs. **2d**, **3d**, or **4d**), but the nature of the hydrophobic substituent does. Thus, in the amide-type conjugates, and according to the data shown in Table 1 for the first series of SMPCs bearing a PEG2 linker, the best inhibition of activity/dimerization was displayed by those bearing the tricyclic lactam (**1d**, ${\mathrm{IC}}_{50}^{\mathrm{act}}$ = 6.7 μM and ${\mathrm{IC}}_{50}^{\dim}$ = 7.8 μM) and the adamantyl (**1f**, ${\mathrm{IC}}_{50}^{\mathrm{act}}$ = 7.6 μM and ${\mathrm{IC}}_{50}^{\dim}$ = 8.1 μM) substituents. Nonetheless, the increased hydrophobicity of **1f** translated into an undesired decrease in solubility. The acridine-conjugated peptide **1e** was also a good dimerization inhibitor (${\mathrm{IC}}_{50}^{\dim}$ = 5.5 μM). The effect of the spacers on *Li*TryR activity/dimerization was assessed by comparing the SMPCs substituted with either a phenyl (**1a**, **2a**, **3a**, and **4a**) or the naphtholactam (**1d**, **2d**, **3d**, and **4d**) and bearing PEG2, PEG1, β-alanyl, and no spacer, respectively. Compounds containing the naphtholactam moiety turned out to be more potent in both assays than the corresponding phenyl derivatives (${\mathrm{IC}}_{50}^{\mathrm{act}}$ = 10.1–6.7 μM vs. 22.3–10.3 μM and ${\mathrm{IC}}_{50}^{\dim}$ = 7.8–5.3 μM vs. 15.0–9.4 μM), and again the length and nature of the spacer were found to be not crucial for activity. Taken together, these results reinforce the superiority of the amphipathic tricyclic lactam for conjugation to **TRL38**.

Regarding SMPCs **7**–**10**, introduction of the aminoalkyl-type linker leads to increased inhibition of the oxidoreductase activity but also to a slight decrease in dimerization disruption (cf. **1a** vs. **7**). Attachment of a second benzyl group to the nitrogen (**8** vs. **7**) has greater impact on dimerization (two-fold increase) than that on enzymatic activity. Different effects on dimerization vs. enzymatic activity have been previously reported to be a consequence of the slow kinetics that have been observed to characterize dimer disruption [36]. Finally, a positive charge at the N is detrimental for dimerization (cf. **7** vs. **9**, and **8** vs. **10**) while the impact on oxidoreductase activity is either lower or nil.

Taken together, our data show that all the hydrophobic SMPCs obtained display significantly improved inhibition of *Li*TryR dimerization and enzymatic activity over the parent **TRL38** peptide prototype. It became apparent that the nature of the hydrophobic group attached to the peptide prototype **TRL38** has a much greater impact on potency than the nature and length of the spacers. Consequently, the SMPCs bearing a naphtholactam (**1d**, **2d**, **3d**, or **4d**) appear to be the best candidates for further studies in the search for novel agents against leishmaniasis.

## 4. Materials and Methods

### 4.1. Instrumentation and Chemicals

Unless otherwise specified, analytical grade solvents and commercially available reagents were used without further purification. Water sensitive reactions were carried out under argon atmosphere. HCTU, DIPEA, Ac_2_O, and protected spacers were purchased from Sigma-Aldrich (Darmstadt, Germany), protected bulky acids were purchased from Fluorochem (Glossop, United Kingdom), and TFA was purchased from Fluka (Germany). Fmoc-protected amino acids were purchased from Fluka, Novabiochem (Merck, Germany), and Iris Biotech (Marktredwitz, Germany). Fmoc-protected Rink amide MBHA resin (0.71 mmol/g loading) was purchased from GS Biochem (China). All amino acids used were of the L configuration.

Target peptides were synthesized using the standard Fmoc/tBu solid-phase orthogonal protection strategy. Compounds were synthesized manually on a 20-position vacuum manifold (Omega, Bienne, Switzerland) connected to a vacuum pump using 20 mL polypropylene syringes (Dubelcco) with a preinserted frit and a teflon stopcock to do the washings and remove the solvents and excess reagents. The coupling reactions were carried out on solid phase using microwave radiation in a Biotage Initiator reactor (Uppsala, Sweden) in a 5 mL vial. Excluding coupling reactions on the microwave reactor, the remaining SPPS reactions were performed, over the previously swollen resin (MBHA polystyrene resin), inside the syringes by adding the corresponding reagents and subsequent automatic stirring on a Grant Bio (POS-300) orbital shaker. After cleavage, the acidic crudes were sedimented in Et_2_O on a Hettlich Universal 320R centrifuge (Sigma-Aldrich ) at 5000 rpm. All the crude and samples were lyophilized using water/acetonitrile mixtures on a Telstar 6–80 instrument. The monitoring of the reactions and final compounds was also performed by HPLC/MS on a HPLC-Waters 12695 connected to a Waters Micromass ZQ spectrometer. The SMPCs were purified by HPFC (high performance flash chromatography) on an SP1 Isolera Biotage instrument using reverse-phase columns (KP-C18-HS 12 g) or on a semipreparative HPLC Waters equipment. As mobile phase, mixtures of A/B were used, where A = 0.05% TFA water and B = acetonitrile with a flow rate of 7 mL/min, using a gradient from 0% of B to 100% of B in 30–45 min and were detected at 217 nm. After purification, SMPCs were lyophilized and dried under reduced pressure in the presence of P_2_O_5_.

The purity of the final SMPCs was checked by an analytical RP-HPLC system on an Agilent Infinity instrument equipped with a diode array and a C18 Sunfire column (4.6 × 150 mm, 3.5 μm). As mobile phase, mixtures of A/B were used, where A = 0.05% TFA water and B = acetonitrile with a flow rate of 1 mL/min. The peptides were analyzed at 214 nm and at 254 nm in a gradient from 2% of B to 100% of B in 15 min. HRMS (EI+) was carried out in an Agilent 6520 Accurate-Mass Q-TOF LC/MS mass spectrometer (Voyager DE-STR Applied Biosystems) using 1% formic acid methanol or methanol/acetonitrile mixtures. 

### 4.2. Syntheses

#### 4.2.1. General Synthetic Protocols

The general peptide elongation procedure involved the following: Fmoc-protected Rink amide MBHA resin was swollen in DCM/DMF/DCM/DMF (3 × 0.5 min), in a filter-equipped syringe. Then, Fmoc removal was performed by treating the resin with a mixture of piperidine/DBU/DMF (1:1:48 in volume) at room temperature (1 × 1 min and 3 × 10 min) and washed with DMF/DCM/DMF/DCM (3 × 0.5 min, each solvent). Later, the free Nα-terminal swollen resin (1 equivalent) was transferred to a 5 mL microwave pressure flask and a solution of the corresponding Fmoc-AA-OH (1.2 equivalents), HCTU (1.2 equivalents), and DIPEA (2.4 equivalents), suspended in dry DMF, were added. The vial was sealed, and the reaction was heated in a microwave vial equipped with a magnetic stirrer, for 10 min at 40 °C. Then, the vial was opened, the supernatant was removed, and new coupling mixture was added. This process was repeated 3 times in total (3 × 10 min) until complete coupling. After the reaction was complete, the resin was transferred to a fritted syringe, drained, washed extensively with (DMF/DCM/DMF/DCM, 5 × 0.5 min), and dried under vacuum. This protocol was repeated for the sequential anchoring of each amino acid until sequence completion. Coupling reactions to primary amines were monitored by the Kaiser ninhydrin test, and to secondary amines by a more sensitive Choranil test. Half-way through the growing sequence, the progress of the reaction was followed by the HPLC-MS analysis of a small sample of peptidyl resin after acidic cleavage.

The general procedure for coupling the spacer and the hydrophobic group to the N-terminus of **TRL38** involved the following: Following a similar procedure to that described for peptide elongation, introduction of both spacers and the final hydrophobic residues was performed by SPPS. The peptidyl resin was swollen (3 × 10 mL each solvent, DCM/DMF/DCM/DMF) in a filter-equipped syringe. Next, the Fmoc protecting group was removed (piperidine/DBU/DMF, 1:1:48 in volume) at room temperature (1 × 1 min and 3 × 10 min) and washed with DMF/DCM/DMF/DCM (3 × 0.5 min, each solvent). Then, the resin was swollen again and treated with the appropriate Fmoc-NH-spacer acid (2 equivalents) or Fmoc-NH-hydrophobic-COOH (2 equivalents) in the presence of HCTU (2 equivalents) and of DIPEA (4 equivalents) in dry DMF (4–5 mL) and were reacted under automatic stirring on a Grant Bio (POS-300) orbital shaker at 320 rpm at room temperature for 2 h. Then, the syringe was attached to the manifold and the excess of reaction mixture was removed by filtration under vacuum. Finally, the resin was washed three times with each solvent and dried under vacuum for 10 min.

After the incorporation of the spacer, and following a similar strategy, the hydrophobic groups at the N-terminus were introduced using the corresponding Fmoc-NH-hydrophobic-COOH (2 equivalents) in each case.

The general procedure for reductive amination involved the following: Peptidyl resin was swollen (3 × 10 mL each solvent, DCM/DMF/DCM/DMF). Then, the Fmoc group was removed upon treating with a solution of DBU/piperidine/DMF, 1:1:48 v:v:v (1 × 1 min and 3 × 10 min). After washing with DCM and DMF twice under vacuum (2 × 0.5 min), the peptidyl resin was transferred to a 10 mL round bottom flask and suspended in 2 mL of anhydrous THF. The flask was sealed with a septum, and then a solution of benzaldehyde (3.6–10 equivalents) in a 1:1 mixture of H_2_O/AcOH (0.15 mL) was added; the reaction mixture was stirred in the orbital shaker for 1 h at 400 rpm. Next, 3.6–10 equivalents of NaBH_3_CN were added and the reaction was stirred (orbital shaking) for 3 h at room temperature. Finally, the reaction mixture was transferred back to a syringe, washed three times with DCM and DMF and dried under vacuum for 10 min.

The procedure for the amine quaternization reaction involved the following: Fmoc-protected swollen resin, placed in a sealed round bottom flask, was treated with an excess of MeI (10 equivalents) and 2,6-lutidine (10 equivalents), the flask was sealed with a septum, and the mixture was shaken overnight at room temperature at 400 rpm. The suspension was filtered in a fritted syringe in the manifold, washed several times with DCM and DMF, and finally dried for 10 min under vacuum.

The general cleavage procedure involved the following: The well-dried functionalized resin-bonded derivatives (1 volume) in a fritted syringe were treated with TFA/TIPS/H_2_O 95:2.5:2.5 (5 volumes) for 4 h at room temperature under 320 rpm orbital shaking. Then, the filtrates were precipitated over cold Et_2_O, in a 50 mL Falcon tube, and centrifuged at 5000 rpm for 10 min at −15 °C. The supernatant was removed, and the process was repeated three times. After supernatant removal, the pellet was suspended in a mixture of water/acetonitrile and freeze-dried. The crudes were purified to give the target SMPCs in high purity.

#### 4.2.2. Solid-Phase Synthesis of Peptide Conjugates 1**a**–**g**, 2**a**,**d**, 3**a**,**d**, 4**a**,**d**, and **7**–**10**

The solid-phase syntheses of the peptide conjugates were as follows: 

Ph-CH_2_-CO-NH-(CH_2_)_2_-O-(CH_2_)_2_-O-CH_2_-CO-NH-PKIIQSVGI-NH_2_ (**1a**): The general protocol for elongation was followed with 0.28 mmol of resin to give Fmoc-peptidyl resin. Next, Fmoc removal from peptidyl resin (0.11 mmol) followed by subsequent Fmoc-PEG2-OH and phenylacetic acid couplings were performed. After cleavage, the crude was purified by reverse-phase chromatography on a Biotage Isolera to give **1a** (156.0 mg, 39% overall yield) as a white lyophilized cotton-like solid. Analytical HPLC (gradient 10–100% of acetonitrile in 10 min), retention time = 6.13 min (99% analytical purity). HRMS (ESI,+) m/z was calculated for C_58_H_97_N_13_O_15_ 1215.72 and found 1215.73 (3.94 ppm). 

Naphthyl-CH_2_-CO-NH-(CH_2_)_2_-O-(CH_2_)_2_-O-CH_2_-CO-NH-PKIIQSVGI-NH_2_ (**1b**): From 0.07 mmol of resin and after elongation, Fmoc-PEG2-OH and naphthyl acetic acid couplings cleavage and purification **1b** was isolated (22.4 mg, 26% overall yield) as a white lyophilized cotton-like solid. Analytical HPLC (gradient 2–100% of acetonitrile in 10 min), retention time = 7.24 min (96% analytical purity). HRMS (ESI,+) m/z was calculated for C_62_H_99_N_13_O_15_ 1266.55 and found 1265.74 (-0.22 ppm).

Biphenyl-CH_2_-CO-NH-(CH_2_)_2_-O-(CH_2_)_2_-O-CH_2_-CO-NH-PKIIQSVGI-NH_2_ (**1c**): The general protocol was followed with 0.07 mmol of resin, Fmoc-PEG2-OH and biphenyl acetic acid, to give, after purification, **1c** (107.7 mg, 42% overall yield) as a white lyophilized cotton-like solid. Analytical HPLC (gradient 2–100% of acetonitrile in 10 min), retention time = 7.17 min (97% analytical purity). HRMS (ESI,+) m/z was calculated for C_64_H_101_N_13_O_15_ 1292.59 and found 1291.75 (3.94 ppm).

(2-(2-oxobenzo[cd]indol-1(*2H*)-yl)-CH_2_-CO-NH-(CH_2_)_2_-O-(CH_2_)_2_-O-CH_2_-CO-NH-PKIIQSVGI-NH_2_ (**1d**): The general protocol was followed with 0.07 mmol of resin, Fmoc-PEG2-OH, and 2-(2-oxobenzo[cd]indol-1(*2H*)-yl)acetic acid, to afford, after purification, **1d** as a white lyophilized cotton-like solid (24.7 mg, 32% overall yield). Analytical HPLC (gradient 10–100 % of acetonitrile in 10 min), retention time = 6.32 min (98% analytical purity). HRMS (ESI,+) m/z was calculated for C_63_H_98_N_14_O_16_ 1307.60 and found 1306.73 (−3.11 ppm).

Acridine-CH_2_-CO-NH-(CH_2_)_2_-O-(CH_2_)_2_-O-CH_2_-CO-NH-PKIIQSVGI-NH_2_ (**1e**): The general protocol was followed with 0.06 mmol of resin, Fmoc-PEG2-OH, and acridine acetic acid. The crude was purified to give **1e** (6.2 mg, 10% overall yield) as a white lyophilized cotton-like solid. Analytical HPLC (gradient 10–100% of acetonitrile in 10 min), retention time = 5.51 min (98% analytical purity). HRMS (ESI,+) m/z was calculated for C_66_H_102_N_14_O_15_ 1330.76 and found 1330.77 (1.30 ppm).

Adamantyl-CH_2_-CO-NH-(CH_2_)_2_-O-(CH_2_)_2_-O-CH_2_-CO-NH-PKIIQSVGI-NH_2_ (**1f**): The general protocol for elongation and spacer/hydrophobic residue couplings was performed with 0.06 mmol of resin, Fmoc-PEG2-OH, and adamantyl acetic acid, to give, after purification, **1f** (4.7 mg, 7% overall yield), as a white lyophilized cotton-like solid. Analytical HPLC (gradient 10–100% of acetonitrile in 10 min), retention time = 7.08 min (95% analytical purity). HRMS (ESI,+) m/z was calculated for C_62_H_107_N_13_O_15_ 1273.80 and found 1273.80 (0.07 ppm).

CH_3_-CH_2_-CO-NH-(CH_2_)_2_-O-(CH_2_)_2_-O-CH_2_-CO-NH-PKIIQSVGI-NH_2_ (**1g**): The general protocol was followed with 0.07 mmol of resin, Fmoc-PEG2-OH, and acetic anhydride. The crude was purified to give **1g**, as a white lyophilized cotton-like solid (14.4 mg, 12% overall yield). Analytical HPLC (gradient 10–100% of acetonitrile in 10 min), retention time = 5.42 min (98% analytical purity). HRMS (ESI,+) m/z was calculated for C_52_H_93_N_13_O_15_ 1139.69 and found 1139.69 (0.69 ppm).

Ph-CH_2_-CO-NH-(CH_2_)_2_-O-CH_2_-CO-NH-PKIIQSVGI-NH_2_ (**2a**): The general protocols for elongation and spacer/hydrophobic residue couplings were performed with 0.12 mmol of resin, Fmoc-PEG1-OH, and phenylacetic acid. The crude was purified to give **2a**, as a white lyophilized cotton-like solid (35 mg, 25% overall yield). Analytical HPLC (gradient 10–95% of acetonitrile in 10 min), retention time = 5.59 min (87% analytical purity). HRMS (ESI,+) m/z was calculated for C_56_H_93_N_13_O_14_ 1171.70 and found 1171.70 (1.33 ppm).

(2-(2-oxobenzo[cd]indol-1(2*H*)-yl)-CH_2_-CO-NH-(CH_2_)_2_-O-CH_2_-CO-NH-PKIIQSVGI-NH_2_ (**2d**): The general protocol was followed with 0.12 mmol of resin, Fmoc-PEG1-OH, and 2-(2-oxobenzo[cd]indol-1(*2H*)-yl)acetic acid. The crude was purified to give **2d** (16.5 mg, 11% overall yield), as a white lyophilized cotton-like solid. Analytical HPLC (gradient 10–95% of acetonitrile in 10 min), retention time = 5.88 min (99% analytical purity). HRMS (ESI,+) m/z was calculated for C_61_H_94_N_14_O_15_ 1262.70 and found 1262.70 (0.58 ppm).

Ph-CH_2_-CO-NH-(CH_2_)_2_-CO-NH-PKIIQSVGI-NH_2_ (**3a**): The general protocol was followed with 0.11 mmol of resin, Fmoc-βalanine-OH, and phenylacetic acid. After purification of the crude **3a** (37 mg, 27% overall yield) was obtained, as a white lyophilized cotton-like solid. Analytical HPLC (gradient 10–100% of acetonitrile in 10 min), retention time = 6.45 min (97% analytical purity). HRMS (ESI,+) m/z was calculated for C_55_H_91_N_13_O_13_ 1145.68 and found 1141.69 (0.29 ppm).

(2-(2-oxobenzo[cd]indol-1(*2H*)-yl)-(CH_2_)_2_-CO-NH-CH_2_-CO-NH-PKIIQSVGI-NH_2_ (**3d**): The general protocol was followed with 0.09 mmol of resin, Fmoc-βalanine-OH, and 2-(2-oxobenzo[cd]indol-1(*2H*)-yl)acetic acid. The crude was purified to give **3d** as a white lyophilized cotton-like solid (4.6 mg, 4% overall yield). Analytical HPLC (gradient 10–100% of acetonitrile in 10 min), retention time = 6.41 min (97% analytical purity). HRMS (ESI,+) m/z was calculated for C_60_H_92_N_14_O_14_ 1232.69 and found 1232.69 (−0.98 ppm).

Ph-CH_2_-CO-NH-PKIIQSVGI-NH_2_ (**4a**): The general protocols for elongation and the hydrophobic residue coupling were performed with 0.09 mmol of resin and phenylacetic acid. The crude was purified to give **4a**, as a white lyophilized cotton-like solid (7.16 mg, 7% overall yield). Analytical HPLC (gradient 10–100% of acetonitrile in 10 min), retention time = 6.86 min (92% analytical purity). HRMS (ESI,+) m/z was calculated for C_52_H_86_N_12_O_12_ 1070.65 and found 1070.65 (2.37 ppm).

(2-(2-oxobenzo[cd]indol-1(2H)-yl)-CH_2_-CO-NH-PKIIQSVGI-NH_2_ (**4d**): The general protocol for elongation and hydrophobic residue coupling were performed with 0.11 mmol of resin and 2-(2-oxobenzo[cd]indol-1(*2H*)-yl) acetic acid. To give, after purification, **4d** as a white lyophilized cotton-like solid (18 mg, 18% overall yield). Analytical HPLC (gradient 10–100% of acetonitrile in 10 min), retention time = 7.38 min (97% analytical purity). HRMS (ESI,+) m/z was calculated for C_57_H_87_N_13_O_13_ 1161.65 and found 1161.66 (0.55 ppm).

Ph-CH_2_-NH-(CH_2_)_2_-O-(CH_2_)_2_-O-CH_2_-CO-NH-PKIIQSVGI-NH_2_ and (Ph-CH_2_)_2_N-(CH_2_)_2_-O-(CH_2_)_2_-O-CH_2_-CO-NH-PKIIQSVGI-NH_2_ (**7** and **8**): Method A: Fmoc-PEG2-peptidil resin (0.03 mmol, RESIN B) was treated with a mixture of piperidine/DBU/DMF (1:1:48 in volume) at room temperature (1 × 1 min and 3 × 10 min) and washed with DMF/DCM/DMF/DCM (3 × 30 s, each solvent) to remove Fmoc group of the spacer. Then, reductive amination protocol was followed using benzaldehyde (3.6 equivalents) and NaBH_3_CN (3.6 equivalents) yielding a mixture of the peptidyl resins **5** and **6**. After cleavage of the mixture, the crude was purified by reverse phase chromatography using semipreparative HPLC (gradient 10–20% of acetonitrile in 30 min). 

From the highest mobility fractions, **7** (10.4 mg, 32% overall yield) was obtained, as a white lyophilized cotton-like solid. Analytical HPLC (gradient 10–100% of acetonitrile in 10 min), retention time = 5.69 min (98% analytical purity). HRMS (ESI,+) *m/z* was calculated for C_57_H_97_N_13_O_14_ 1188.48 and found 1187.73 (3.76 ppm).

From the lowest mobility fractions, 3.2 mg (9% overall yield) of compound **8** was obtained, as a white lyophilized cotton-like solid. Analytical HPLC (gradient 10–100% of acetonitrile in 10 min), retention time = 6.47 min (95% analytical purity). HRMS (ESI,+) *m/z* was calculated for C_64_H_103_N_13_O_14_ 1277.77 and found 1277.78 (2.95 ppm). 

Method B: A similar protocol to that described in method A was followed with 0.073 mmol of Fmoc-PEG2-peptidil resin, which after removal of Fmoc group was reacted with benzaldehyde (10 equivalents) and NaBH_3_CN (10 equivalents), to give, after cleaving and purification by reverse phase on a Biotage Isolera (0–100%, acetonitrile), compound **8** (20 mg, 21% overall yield).

(Ph-CH_2_)(Me)_2_N^+^-(CH_2_)_2_-O-(CH_2_)_2_-O-CH_2_-CO-NH-PKIIQSVGI-NH_2_ I^−^ and (Ph-CH_2_)_2_(Me)N^+^-(CH_2_)_2_-O-(CH_2_)_2_-O-CH_2_-CO-NH-PKIIQSVGI-NH_2_ I^−^ (**9** and **10**): The mixture of peptidyl resins **5** and **6** (0.073 mmol) was quaternized, following the general protocol, by reaction with MeI in the presence of 2,6-lutidine. After cleavage, the crude was purified by semipreparative HPLC (gradient 10–20% of acetonitrile in 30 min). From the highest mobility fractions, the dimethylated compound **9** was isolated (22 mg, 25% overall yield), as a white lyophilized cotton-like solid. Analytical HPLC (gradient 10–100% of acetonitrile in 10 min), retention time = 5.70 min (99% analytical purity). HRMS (ESI, +) m/z was calculated for C_59_H_102_N_13_O_14_^+^ 1217.54 and found 1216.77 (−1.93 ppm).

From the lowest mobility fractions, compound **10** (7.9 mg, 8% overall yield) was isolated, as a white lyophilized cotton-like solid. Analytical HPLC (gradient 10–100% of acetonitrile in 10 min), retention time = 6.33 min (98% analytical purity). HRMS (ESI,+) *m/z* was calculated for C_65_H_106_N_13_O_14_^+^ 1293.64 and found 1292.80 (−4.56 ppm). 

### 4.3. Molecular Modeling and Computer Simulations

Structural cavities in *Li*TryR dimer and monomer were calculated using the Computed Atlas of Surface Topography of proteins (CASTp) web server [32] and a probe radius of 1.4 Å. Molecular dynamics simulations were run in the AMBER force field, as described previously for *Li*TryR dimer and complexes [22,23,24,25,26,27,28]. The molecular graphics program PyMOL (v. 1.8, Schrödinger, 2015) was employed for molecular editing, visualization, and figure preparation.

### 4.4. Biological Evaluation

#### 4.4.1. *Li*TryR Purification

The recombinant versions of *Li*TryR were purified, according to the protocol described by Toro et al. [22]. Briefly, the pRSETA-HIS-*Li*TryR construct was transformed either alone or in combination with the pET24a-FLAG-*Li*TryR construct into BL21 (DE3) Rossetta 2 *E. coli* strain. An overnight culture of *E. coli* cells grown at 37 °C in LB medium with suitable antibiotics and vigorous shaking was diluted (1:100) in the same culture medium and allowed to grow in the same conditions until the OD_600_ reached 0.5. At this point, *Li*TryR expression was induced with 1 mM isopropyl-β-D-1-thiogalactopyranoside (Thermo Fisher Scientific, Waltham, MA, USA) for 16 h at 26 °C. The *E. coli* cells were harvested by centrifugation at 9000× *g* and 4 °C for 5 min. The wet pellets were resuspended in a lysis buffer containing 50 mM Tris pH 7, 300 mM NaCl, 25 mM imidazole, 1 mg/mL lysozyme, and a protease inhibitor cocktail. Following a 30 min incubation on ice, the cell lysate was disrupted by sonication on ice (50% pulses, potency 7) for 30 min using a Sonifier Cell Disruptor B15 (Branson, Danbury, CT, USA) and clarified by centrifugation at 50,000× *g* and 4 °C for 1 h. The supernatant containing the soluble protein fraction was collected and sonicated again, as previously described for 10 min. The supernatant was filtered using a low protein binding PVDF filter with a pore size of 0.22 µm (Merck Millipore, Burlington, MA, USA) and subsequently loaded onto a HisTrap column (GE Healthcare, Chicago, IL, USA) for 16 h at 4 °C using a P-1 peristaltic pump (GE Healthcare, Chicago, IL, USA). Once loaded, the HisTrap column was connected to an ÄKTA purifier UPC 10 (GE Healthcare, Chicago, IL, USA) and extensively washed with a 10% gradient between buffers A (50 mM Tris pH 7 and 300 mM NaCl) and B (50 mM Tris pH 7, 300 mM NaCl, and 500 mM imidazole). Soluble *Li*TryR was eluted with a 40% gradient between buffers A and B. The yellow fractions containing recombinant *Li*TryR were pooled and subsequently loaded onto a HiPrep 26/10 Desalting column (GE Healthcare, Chicago, IL, USA), previously equilibrated with buffer A. Finally, *Li*TryR-containing fractions were pooled again and concentrated to 2 mg/mL using an Amicon^®^ Ultra-15 50K filter (Merck Millipore, Burlington, MA, USA) and an equal volume of glycerol was added before storing at −20 °C. 

#### 4.4.2. *Li*TryR Oxidoreductase Activity Assay

*Li*TryR oxidoreductase activity was assessed spectrophotometrically using a modified version of the DTNB-coupled assay, described by Hamilton et al. [35]. Briefly, reactions were performed at 26 °C in 250 μL of HEPES buffer (pH 7.5, 40 mM) containing EDTA (1 mM), NADP^+^ (30 μM), DTNB (25 μM), TS_2_ (1 μM), NADPH (150 μM), glycerol (0.02%), DMSO (1.75%), and recombinant HIS-*Li*TryR (7 nM). DTNB, glycerol, and DMSO concentrations used in this assay do not have any relevant effect on the kinetics of the *Li*TryR oxidoreductase reaction. Reactions were started by addition of a mixture (50 μL/well) of the substrates (NADPH and TS_2_).

*Li*TryR oxidoreductase activity was monitored at 26 °C by an increase in absorbance at 412 nm in an EnSpire Multimode Plate Reader (PerkinElmer, Waltham, MA, USA). In this DTNB-coupled assay, one molecule of T(SH)_2_ reduces one molecule of DTNB, producing two TNB molecules. The TNB concentration was obtained by multiplying the absorbance values by 100 (50 μM TNB generates 0.5 arbitrary units of absorbance at 412 nm). 

For IC_50_ determinations, the initial velocities were calculated from the slope of the initial linear part of the reaction curves. The initial velocities of the reaction progress curves in the presence of inhibitor (concentrations ranging from 0.1 to 25 μM) were normalized as a percentage with respect to the initial velocity in the presence of the inhibitor vehicle (DMSO). IC_50_ values were obtained by fitting these percentages to a nonlinear regression model with GraFit 6 software (Erithacus, Horley, Surrey, U.K.). All the assays were performed in triplicate. 

#### 4.4.3. *Li*TryR Dimer Quantitation Assay

The relative amount of the *Li*TryR dimeric form in the presence of the SMPCs was assessed using an enzyme-linked immunosorbent assay (ELISA), developed in our laboratory [22]. Briefly, HIS-FLAG-tagged *Li*TryR (400 nM) was incubated in dimerization buffer (1 mL of 50 mM Tris pH 8.0 and 300 mM NaCl) for 16 h at 37 °C with vigorous shaking and in a humid atmosphere in the presence of the different compounds ranging from 25 to 3.1 μM. Following a 15 min centrifugation at 18,000× *g* and room temperature, the supernatants were added to the α-FLAG-coated plates (200 μL/well) and incubated for 30 min at 37 °C with vigorous shaking in a humid atmosphere. The plates were washed five times with TTBS buffer (2 mM Tris pH 7.6, 138 mM NaCl, and 0.1% Tween-20) and incubated with diluted monoclonal α-HIS horseradish peroxidase (HRP)-conjugated antibody (200 μL/well, 1:50,000, Abcam, Cambridge, UK) in fatty acid- and essentially globulin-free bovine serum albumin (5%) prepared in TTBS buffer for 1 h at room temperature. The plates were washed again five times and the *o*-phenylenediamine dihydrochloride substrate, prepared according to the manufacturer’s instructions, was added (100 μL/well). The enzymatic reactions were stopped after 5 min by addition of 0.5 μM H_2_SO_4_ (100 μL/well) and the absorbances were measured at 490 nm in an EnSpire Multimode Plate Reader (PerkinElmer, Waltham, MA, USA). 

For IC_50_ determinations, the absorbance values in the presence of different concentrations of inhibitor were normalized as a percentage with respect to the absorbance value in the presence of DMSO. The IC_50_ values were obtained by fitting these percentages to a nonlinear regression model with GraFit 6 software (Erithacus, Horley, Surrey, U.K.). The assays were carried out in triplicate and repeated three times.

## Figures and Tables

**Table 1:** Half-maximal inhibitory concentration (IC_50_) ± standard error (SE) values for **TRL38** conjugates in the *Li*TryR oxidoreductase activity and dimer quantitation assays ^a^.

Peptide	R	Spacer Type	$IC_{50}^{\mathrm{act}}\;(\mu M){\;}^{c}$	$IC_{50}^{\dim}\;(\mu M){\;}^{d}$
**TRL38 ^b^**			>25 (65.1%) ^e,^*	>25 (61.4%) ^e,&^
Amide Link				
**1a**	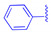	-CH_2_-CH_2_-O-CH_2_-CH_2_-O-CH_2_-CO-	22.0 ± 0.7	9.4 ± 0.1
**1b**	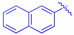	-CH_2_-CH_2_-O-CH_2_-CH_2_-O-CH_2-_CO-	18.2 ± 1.0	8.7 ± 0.5
**1c**	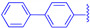	-CH_2_-CH_2_-O-CH_2_-CH_2_-O-CH_2_-CO-	16.6 ± 3.8	22.9 ± 1.4
**1d**	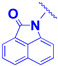	-CH_2_-CH_2_-O-CH_2_-CH_2_-O-CH_2_-CO-	6.7 ± 0.4	7.8 ± 0.1
**1e**	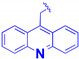	-CH_2_-CH_2_-O-CH_2_-CH_2_-O-CH_2_-CO-	14.6 ± 3.8	5.5 ± 0.5
**1f**	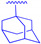	-CH_2_-CH_2_-O-CH_2_-CH_2_-O-CH_2_-CO-	7.6 ± 1.2	8.1 ± 0.2
**3g**	H	-CH_2_-CH_2_-O-CH_2_-CH_2_-O-CH_2_-CO-	13.4 ± 1.7	21.1 ± 1.6
**2a**	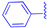	-CH_2_-CH_2_-O-CH_2_-CO-	22.3 ± 1.5	15.0 ± 0.6
**2d**	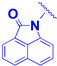	-CH_2_-CH_2_-O-CH_2_-CO-	6.9 ± 1.9	7.1 ± 0.1
**3a**	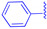	-CH_2_-CH_2_-CO-	20.6 ± 1.6	12.7 ± 1.1
**3d**	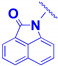	-CH_2_-CH_2_-CO-	10.1 ± 1.5	5.3 ± 0.9
**4a**	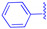	No spacer	10.3 ± 1.8	11.4 ± 0.1
**4d**	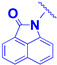	No spacer	8.0 ± 1.5	7.1 ± 0.2
		Aminoalkyl link		
**7**	Ph-CH_2_N	-CH_2_-CH_2_-O-CH_2_-CH_2_-O-CH_2_-CO-	8.3 ± 1.4	12.6 ± 0.6
**8**	(Ph-CH_2_)_2_N	-CH_2_-CH_2_-O-CH_2_-CH_2_-O-CH_2_-CO-	9.8 ± 2.3	7.1 ± 0.5
**9**	Ph-CH_2_N^+^(Me)_2_	-CH_2_-CH_2_-O-CH_2_-CH_2_-O-CH_2_-CO-	18.3 ± 2.6	>25 (53.3 %) ^&^
**10**	(Ph-CH_2_)_2_N^+^Me	-CH_2_-CH_2_-O-CH_2_-CH_2_-O-CH_2_-CO-	9.2 ± 1.1	21.2 ± 3.5

^a^ Results are representative of three independent experiments, each performed in triplicate. ^b^ Linear peptide **TRL38** is included as the reference compound. ^c^ Enzymatic activity according to a modified assay described by Hamilton et al. [35]. ^d^ Dimer quantitation ELISA [22]. ^e^ Percentage of inhibition observed at 25 μM (reliable IC_50_ values could not be determined in this case). Previously reported IC_50_ values were obtained after preincubation of *Li*TryR with TRL38 for 10 min [22]. The values shown in this table were obtained without prior incubation due to the observed time dependence of the inhibitory process [36]. * *Li*TryR oxidoreductase activity at 25 μM (%). ^&^ *Li*TryR dimer at 25 μM (%).

## Data Availability

Data is contained within the article.
